# State-Dependent and Trait-Like Characteristics of Dysfunctional Attitudes in Patients With Major Depressive Disorder

**DOI:** 10.3389/fpsyt.2020.00645

**Published:** 2020-07-10

**Authors:** Bangshan Liu, Jinrong Sun, Xuemei Qin, Mi Wang, Xiaowen Lu, Qiangli Dong, Liang Zhang, Jin Liu, Yumeng Ju, Ping Wan, Hua Guo, Futao Zhao, Yan Zhang, Lingjiang Li

**Affiliations:** ^1^ Department of Psychiatry, The Second Xiangya Hospital, Central South University, Changsha, China; ^2^ Mental Health Institute of Central South University, China National Clinical Research Center on Mental Disorders (Xiangya), China National Technology Institute on Mental Disorders, Hunan Technology Institute of Psychiatry, Hunan Key Laboratory of Psychiatry and Mental Health, Changsha, China; ^3^ Affiliated WuTaiShan Hospital of Medical College of Yangzhou University, Yangzhou Mental Health Centre, Yangzhou, China; ^4^ Department of Psychiatry, Zhumadian Psychiatric Hospital, Zhumadian, China

**Keywords:** major depressive disorder, dysfunctional attitudes, state-dependent, trait-like, longitudinal

## Abstract

**Background:**

Studies have shown that patients with major depressive disorder (MDD) exhibit elevated dysfunctional attitudes (DAs). However, it remains controversial whether the DAs are state-dependent or trait-like features of MDD.

**Methods:**

This study recruited 172 patients and 159 healthy controls (HCs) at baseline. DAs were respectively assessed by the 24-item Hamilton Depression Rating Scale (HAMD_24_) and the Chinese version of Dysfunctional Attitude Scale form A (C-DAS-A). After baseline, patients received a 6-month antidepressant treatment. General linear models were used to analyze the differences in the C-DAS-A total and factor scores between the acute and remitted MDD groups and the HC group. Paired t tests were used to assess the changes of C-DAS-A total and factor scores in the remitted MDD group before and after treatment.

**Results:**

At baseline, patients with MDD showed significantly higher scores in C-DAS-A and its subscales than HCs (all *P* < 0.05). After treatment, the C-DAS-A total and factor scores decreased significantly in the remitted MDD group (all P < 0.05). However, the C-DAS-A total (*P* = 0.005) and five factors’ scores (vulnerability, attraction and repulsion, perfectionism, compulsion, and dependence) remained elevated in the remitted MDD group as compared with HCs (all P < 0.05). There were moderate correlations between the baseline and remission phase C-DAS-A total and five factors’ scores (all P < 0.05).

**Conclusion:**

DAs show a mixture of state-dependent and trait-like characteristics in MDD with partial improvement in the remission phase. Special attention should be paid to the residual DAs in the remitted MDD for the prevention of relapse.

## Introduction

Distorted and maladaptive cognitive beliefs are commonly seen in patients with major depressive disorder (MDD). These beliefs, usually developed in the early childhood, have been recognized as predisposing and perpetuating factors of MDD. The maladaptive cognitive beliefs are usually latent and not observable, but can become manifest in response to stress and influence one’s perception, appraisal, and behavior. Maladaptive cognitive beliefs can also promote the formation of dysfunctional attitudes (DAs), which are counterproductive cognitions about the self, others, and the future. In accordance with Beck’s cognitive theory, DAs constitute important components of cognitive vulnerability to depression and are associated with increased risk of chronicity and relapse in patients with MDD (1).

To date, studies have reported that DAs predict increased risk of onset, persistence, and recurrence of depression (2–6). However, it remains controversial whether the DAs are state-dependent or trait-like features of MDD. Some studies found that the DAs would covary with depression severity and disappear in the remission phase of MDD (7–10); while other studies found that the DAs persist in patients with MDD even after effective treatment (11, 12). To address the discrepancy among different studies, Zuroff et al. put forward a state-trait model of DAs (12), which states that DAs would exhibit both state-dependent and trait-like characteristics in patients with MDD. Specifically, the DAs would covary with the depression severity but would also maintain relative stability across the acute and remission phases of MDD.

Herein we report a study examining the Zuroff’s state-trait model of DAs in a Chinese sample of patients with MDD. We longitudinally assessed and analyzed the DAs in patients with MDD from the acute phase to the remission phase. We hypothesized that patients with MDD would exhibit DAs in the acute phase and the DAs would be partially (not fully) improved in the remission phase.

## Materials and Methods

### Participants

The data of this study comes from a longitudinal project which aims to investigate the biological and psychological mechanisms of MDD (Project name: *Hypothalamic–pituitary–adrenal axis function and magnetic resonance imaging study of trauma-related depression*. Registration number: ChiCTR1800014591). One hundred and seventy-two adult patients with MDD were recruited from the Zhumadian Psychiatric Hospital, Henan, China. The diagnoses of the patients were confirmed by two trained psychiatrists with the Structured Clinical Interview for Diagnostic and Statistical Manual of Mental Disorders, Fourth Edition, Text Revision (DSM-IV-TR). Patients should exhibit at least moderate depression with a total score of the 24-item Hamilton rating scale for depression (HAMD_24_) ≥ 20 at baseline. The exclusion criteria for MDD patients were: 1) age ≥56 or ≤17 years; 2) accepted psychiatric medication within two weeks (six weeks for fluoxetine); 3) had any other psychiatric disorders apart from generalized anxiety disorder; 4) had any major physical condition. One hundred and fifty-nine healthy controls (HCs) with HAMD_24_ total score ≤ 7 were recruited from local community of Zhumadian, Henan, China. The detailed inclusion and exclusion criteria for subject recruitment had been reported in our previous paper (13).

After the baseline assessment, all patients were treated by their attending psychiatrists with a mainstream antidepressant, either a selective serotonin reuptake inhibitor or a serotonin-norepinephrine reuptake Inhibitor. Patients were assessed with HAMD_24_ at the end of the 0.5, 1^st^, 2^nd^, 3^rd^, 4^th^, 5^th^ and 6^th^ months. Clinical remission was defined as HAM-D_24_ ≤ 7 in two consecutive months and later time points during the follow-up period. Ninety-two patients were followed up at the end of the sixth month, among which 74 patients were remitted (rMDD subgroup) and 18 were unremitted. Since our study focused on the state and trait-like characteristics of DAs in MDD, the analysis of follow-up data was limited to the rMDD subgroup.

### Assessment of DAs

As reported in our previous paper (14), the DAs were assessed by the Chinese version of DAS form A (C-DAS-A). The C-DAS-A was translated from the Dysfunctional Attitude Scale (15) by Chen et al. in 1998 (16). In their construct validity analysis, Chen et al. (16) proposed an eight-factor structure (vulnerability, attraction and repulsion, perfectionism, compulsion, seeking applause, dependence, self-determination attitude, and cognition philosophy) of the C-DAS-A, which was widely adopted in the later studies, also adopted in present study. For a detailed description about the characteristics of the C-DAS-A, please refer to our previous paper (14).

### Statistical Analyses

Two-sample independent t tests and Chi-square tests were selected to analyze the demographic information between the MDD and HC groups at baseline. General linear models were used to assess the differences in C-DAS-A total and factor scores between MDD or rMDD subgroups and HCs with unmatched demographic variables controlled. Paired t-tests were performed to analyze the differences in C-DAS-A total and factor scores in the rMDD subgroup before and after treatment. Pearson correlation analyses were applied to assess the correlations between the baseline and remission phase C-DAS-A total and factor scores and between the changes of HAMD_24_ and changes of C-DAS-A total and factor scores before and after treatment in the rMDD subgroup. Statistical significance was set as a two-tailed P ≤ 0.05.

## Results

### Demographic and Clinical Information of the MDD and HC Groups

The demographic and clinical characteristics of the included subjects were displayed in **Table 1**. There was no statistical significance in age and sex at baseline between the MDD and HC groups (*P* > .05). The HC group showed higher education years and BMI than the MDD group (*P* < .05). The MDD group showed significantly higher HAMD_24_ and C-DAS-A scores than the HC group at baseline (*P* < 0.001). There was no significant difference between the rMDD subgroup and MDD group in demographic and clinical information (**Table 1**).

**Table 1:** Demographic and clinical information by groups.

	Baseline			MDD *vs.* HC		MDD *vs.* rMDD	
	MDD	HC	rMDD	t or x^2^	*P*	t or x^2^	*P*
	(n = 172)	(n = 159)	(n = 74)				
Age (years)	35.11 ± 9.67	34.55 ± 9.12	36.26 ± 10.23	0.538	0.591	-0.838	0.403
Female (%)	97 (56.4%)	85 (53.5%)	42(56.8%)	0.288	0.592	0.003	0.958
Education (years)	10.26 ± 3.48	11.10 ± 3.59	10.89 ± 3.39	−2.157	**0.032**	−1.312	0.191
BMI (kg/m^2^)	22.01 ± 3.06	23.65 ± 3.00	22.24 ± 2.93	−4.885	**0.000**	-0.549	0.583
HAMD_24_	31.63 ± 7.31	1.40 ± 1.77	31.93 ± 7.33	52.559	**0.000**	−0.299	0.765
C-DAS-A	155.74 ± 28.07	125.21 ± 26.10	156.08 ± 28.92	10.225	**0.000**	−0.086	0.932

data are presented as Mean ± SD; MDD, major depressive disorder; HC, healthy control; BMI, Body mass index; HAMD_24_, 24-item Hamilton Rating Scale for Depression; C-DAS-A, Chinese version of dysfunctional attitude scale-form A. Bold values indicate statistical significance.

### Comparison of the C-DAS-A Total and Factor Scores in the MDD and HC Groups

The C-DAS-A total and factor scores of patients with MDD and HCs were shown in **Table 2**. MDD patients scored higher than HCs in C-DAS-A total and eight factor scores at baseline (all *P* < .001).

**Table 2:** Comparison of the C-DAS-A total and factor scores in the MDD and HC groups.

Item	MDD (n = 172)	HC (n = 159)	F	*P*
Total score	155.74 ± 28.07	125.21 ± 26.10	94.49	0.000
**Factor scores**				
vulnerability	18.08 ± 4.61	14.75 ± 4.51	41.45	**0.000**
attraction and repulsion	18.35 ± 5.74	12.86 ± 4.99	80.09	**0.000**
perfectionism	18.76 ± 5.67	14.87 ± 5.02	43.92	**0.000**
compulsion	18.99 ± 4.60	16.25 ± 3.98	28.83	**0.000**
seeking applause	19.55 ± 5.41	16.13 ± 6.77	21.64	**0.000**
dependence	19.62 ± 4.79	15.29 ± 4.62	65.91	**0.000**
self-determination attitude	22.17 ± 5.65	17.82 ± 5.09	53.55	**0.000**
cognition philosophy	20.21 ± 5.38	17.25 ± 5.03	16.89	**0.000**

education and BMI were controlled as covariates in the general linear model analyses; data are presented as Mean ± SD; C-DAS-A, Chinese version of dysfunctional attitude scale-form A; MDD. major depressive disorder; HC, healthy control.

Bold values indicate statistical significance.

### Comparison of the C-DAS-A Total and Factor Scores in the rMDD Subgroup and the HC Group

The C-DAS-A total and factor scores of the rMDD subgroup in the remission phase and HCs were shown in **Table 3**. rMDD patients showed more DAs in vulnerability, attraction and repulsion, perfectionism, compulsion and dependence than HCs in the remission phase (all P < 0.01).

**Table 3:** Comparison of the C-DAS-A total and factor scores in the rMDD subgroup and the HC group.

	rMDD (n = 74)	HC (n = 159)	F	*P*
Total score	137.65 ± 30.79	125.21 ± 26.10	9.64	0.002
**Factor scores**				
vulnerability	16.61 ± 5.00	14.75 ± 4.51	8.43	**0.004**
attraction and repulsion	15.26 ± 5.36	12.86 ± 4.99	9.95	**0.002**
perfectionism	16.99 ± 6.06	14.87 ± 5.02	8.95	**0.003**
compulsion	17.73 ± 5.39	16.25 ± 3.98	6.91	**0.009**
seeking applause	17.20 ± 5.03	16.13 ± 6.77	1.03	0.312
dependence	17.22 ± 4.74	15.29 ± 4.62	8.50	**0.004**
self-determination attitude	19.00 ± 6.31	17.82 ± 5.09	2.40	0.112
cognition philosophy	18.16 ± 4.88	17.25 ± 5.03	0.49	0.487

education and BMI were controlled as covariates in the general linear model analyses; data are presented as Mean ± SD; C-DAS-A, Chinese version of dysfunctional attitude scale-form A; MDD, major depressive disorder; HC, healthy control.

Bold values indicate statistical significance.

### Comparison of the C-DAS-A Total and Factor Scores in the rMDD Subgroup Before and After Treatment

The comparison of the C-DAS-A total and factor scores before and after treatment in the rMDD subgroup was shown in **Table 4**. Significant reductions were observed in the C-DAS-A total and eight factor scores in the remission phase (all P < 0.05).

**Table 4:** Comparison of the C-DAS-A total and factor scores in the rMDD subgroup before and after treatment.

Item	Baseline	Remission	t	*P*
Total score	156.08 ± 28.92	137.65 ± 30.79	4.862	0.000
**Factor scores**				
Vulnerability	17.85 ± 4.19	16.61 ± 5.00	2.324	**0.023**
Attraction and repulsion	18.95 ± 5.44	15.26 ± 5.36	4.639	**0.000**
Perfectionism	18.97 ± 5.68	16.99 ± 6.06	2.661	**0.010**
Compulsion	19.20 ± 4.64	17.73 ± 5.39	2.001	**0.049**
Seeking applause	19.09 ± 5.39	17.20 ± 5.03	2.643	**0.010**
Dependence	19.77 ± 4.97	17.22 ± 4.74	3.720	**0.000**
Self-determination attitude	22.08 ± 6.53	19.00 ± 6.31	3.510	**0.001**
Cognition philosophy	20.16 ± 5.53	18.16 ± 4.88	2.511	**0.014**

data are presented as Mean ± SD; C-DAS-A, Chinese version of dysfunctional attitude scale-form A; MDD, major depressive disorder. Bold values indicate statistical significance.

### Correlations Between the Baseline and Remission Phase C-DAS-A Total and Factor Scores, Between the Changes of HAMD_24_ and Changes of C-DAS-A Total and Factor Scores Before and After Treatment in the rMDD Subgroup

The correlations between the baseline and remission phase C-DAS-A total and factor scores in the rMDD subgroup were shown in **Table 5**. Moderate correlations were observed in the C-DAS-A and five factors (vulnerability, perfectionism, seeking applause, dependence and self-determination attitude) scores (all P < 0.05) before and after treatment. The correlations between the changes of HAMD_24_ and changes of C-DAS-A total and factor scores before and after treatment in the rMDD subgroup were shown in **Table 6**. Only a mild correlation between the change of HAMD_24_ and the change of seeking applause was observed.

**Table 5:** Correlations between the baseline and remission phase C-DAS-A total and factor scores in the rMDD subgroup.

	Total score	vulnerability	attraction and repulsion	perfectionism	compulsion	seeking applause	dependence	self-determination attitude	cognition philosophy
r	0.405	0.511	0.197	0.403	0.208	0.303	0.260	0.309	0.138
*P*	**0.000**	**0.000**	0.092	**0.000**	0.075	**0.009**	**0.025**	**0.007**	0.241

C-DAS-A, Chinese version of dysfunctional attitude scale-form A; rMDD, remitted major depressive disorder. Bold values indicate statistical significance.

**Table 6:** Correlations between the changes of HAMD_24_ and changes of C-DAS-A total and factor scores before and after treatment in the rMDD subgroup.

	DAS total score	vulnerability	attraction and repulsion	perfectionism	compulsion	seeking applause	dependence	self-determination attitude	cognitionphilosophy
r	0.124	0.016	0.141	0.004	0.203	0.297	0.145	−0.008	−0.010
*P*	0.294	0.293	0.231	0.972	0.083	**0.010**	0.217	0.948	0.930

C-DAS-A, Chinese version of dysfunctional attitude scale-form A; rMDD, remitted major depressive disorder. Bold values indicate statistical significance.

## Discussion

In this study, we investigated the DAs in a sample of Chinese patients of MDD from the acute phase to the remission phase. Our results revealed pervasive DAs in all of the eight factors in acute MDD. With effective treatment, all of the eight factor scores were decreased significantly, while the factors of vulnerability, attraction and repulsion, perfectionism, seeking applause, and dependence remained elevated in the remission phase of MDD. The C-DAS-A total factor scores in the remission phase were moderately correlated with that in the acute phase in MDD. Our results indicate that the DAs have both the state-dependent and trait-like features, consistent with Zuroff’s state-trait vulnerability model of DAs in MDD (12).

Our results revealed a significant decrease in the C-DAS-A total and factor scores in remitted MDD patients after treatment, which is consistent with previous studies reporting significant improvement of DAs with antidepressant treatment (5, 17). The fluctuation of DAs across different phases of depression supports a state-dependent characteristic of DAs in MDD. In the acute phase of MDD, due to the intact relationship between emotion and cognition, patients would exhibit more DAs under the impact of the depressed mood. For example, low mood in depression may comparatively reflect on DAs such as “If others dislike you, you cannot be happy” or “It is difficult to be happy unless one is good looking, intelligent, rich and creative”. With the improvement of the mood status in the remission phase, the impact of low mood on cognition would be eliminated, showing as decreased DAs.

However, despite the significant improvement in the DAs in the remission phase of MDD, the total and five factors (vulnerability, attraction and repulsion, perfectionism, compulsion, and dependence) scores of C-DAS-A in the rMDD subgroup remained elevated as compared with HCs. The residual DAs in the remission phase suggest a trait-like characteristic of DAs in MDD, which is consistent with previous studies reporting elevated DAS total and factor scores in patients with MDD even after effective treatment (11, 17, 18). Our results also revealed a moderate correlation between the baseline and follow-up C-DAS-A total and factor scores and rare correlations between the changes of HAMD_24_ and changes of C-DAS-A total and factor scores before and after treatment in the rMDD subgroup, indicating that the DAs are relatively stable across different phases of MDD, which further supported the trait-like characteristic of DAs in MDD.

The findings of mixed state-dependent and trait-like characteristics of DAs in MDD are both theoretically and clinically meaningful. From the theoretical perspective, our results supported a bidirectional relationship between DAs and depression, which means that DAs would increase the risk of depression and depression would inversely bring about more DAs. The vicious cycle between depression and DAs may help explain the increased risk of depressive episodes among those with a history of depression. From the clinical perspective, the state-dependent characteristic of DAs calls for rapid and effective pharmacological interventions to improve mood in patients with acute depression, so as to break the vicious cycle between DAs and depression. Moreover, psychological interventions perturbating the DAs are also helpful for alleviating depression, augmenting and strengthening the effect of pharmacotherapy, and reducing the risk of relapse. The trait-like characteristic of DAs in MDD also provides theoretical basis for taking DAS as a tool in identifying subjects vulnerable to MDD. For those with no clinical depression but showing elevated DAs, early psychological interventive strategies may be adopted to reduce the risk of depression in the future.

## Limitations

There are some limitations in our study which raised caution in interpreting the results. First, there was a high dropout rate in the follow-up period, resulting in a small number of unremitted patients at the end of 6 months. However, we believe that the bias attributed to the high dropout rate was low as there was no significant difference between the rMDD subgroup and the total MDD group in demographic information. Second, the duration of follow-up is limited to 6 months. We are unable to know whether the residual DAs would persist in a longer time span and predict an increased risk of relapse in MDD. Future studies are needed to clarify the trajectory of DAs in MDD in a longer time period.

## Conclusions

DAs show a mixture of state-dependent and trait-like characteristics in patients with MDD. Attention should be paid to the residual DAs in the remission phase of MDD. Continuous psychological interventions are suggested for rMDD patients. In addition, early interventions for the subjects with DAs may be an effective strategy for the prevention of depression.

## Data Availability Statement

The raw data supporting the conclusions of this article available on request to the corresponding author.

## Ethics Statement

The studies involving human participants were reviewed and approved by the Ethics Committee of the Second Xiangya Hospital of Central South University and the Ethics Committee of the Zhumadian Psychiatric Hospital. The patients/participants provided their written informed consent to participate in this study.

## Author Contributions

LL and YZ codesigned the topic. BL, JS, MW, XL, QD, LZ, JL, YJ, PW, HG, FZ, and YZ are responsible for participant recruitment and data collection. BL and XQ undertook the statistical analyses. JS wrote the initial draft of the manuscript. BL contributed substantial revisions to the manuscript. All authors have approved the final version of this manuscript.

## Funding

This study was supported by the National Science and Technologic Program of China (2015BAI13B02), the Defense Innovative Special Region Program (17-163-17-XZ-004-005-01), the National Natural Science Foundation of China (81171286, 91232714, and 81601180). The funding sources had no role in the study design, data collection and analysis, interpretation of the data, preparation and approval of the manuscript, and decision to submit the manuscript for publication.

## Conflict of Interest

The authors declare that the research was conducted in the absence of any commercial or financial relationships that could be construed as a potential conflict of interest.
